# Identification of the pan-allergen tropomyosin from the common bed bug (*Cimex lectularius*)

**DOI:** 10.1038/s41598-024-57877-3

**Published:** 2024-03-27

**Authors:** Johnalyn M. Gordon, Zachary C. DeVries

**Affiliations:** Department of Entomology, University of Kentucky, Lexington, KY USA

**Keywords:** Entomology, Risk factors

## Abstract

Tropomyosin is a muscle contraction protein documented across all animal life. Despite its ubiquity, its unique structure in invertebrates leads to allergic responses in humans that vertebrate tropomyosin does not. High degrees of homology can explain cross-reactivity between tropomyosin derived from distantly-related arthropod species and establishes tropomyosin as a panallergen. Given this cross-reactivity and that they are commonly found in high numbers indoors, research on the potential of the common bed bug (*Cimex lectularius* L.) to contribute tropomyosin to the indoor environment is needed. Therefore, we investigated tropomyosin homology between bed bugs and known tropomyosin allergens from other taxa, tropomyosin in bed bug bodies, feces, and exuviae (cast skins), tropomyosin persistence over time, and impacts of common bed bug treatment strategies on detectable tropomyosin. Tropomyosin was detected in mechanically fractured bed bug cadavers and was detectable in bed bugs cadavers aged for 18 months. Additionally, a survey of pest management professionals showed dead bed bugs are not cleaned up following treatment. As such, dead bed bugs could act as tropomyosin reservoirs following bed bug treatment and exposure to tropomyosin from bed bugs could sensitize individuals and lead to increased responses to other arthropod tropomyosin.

## Introduction

**Figure 1:**
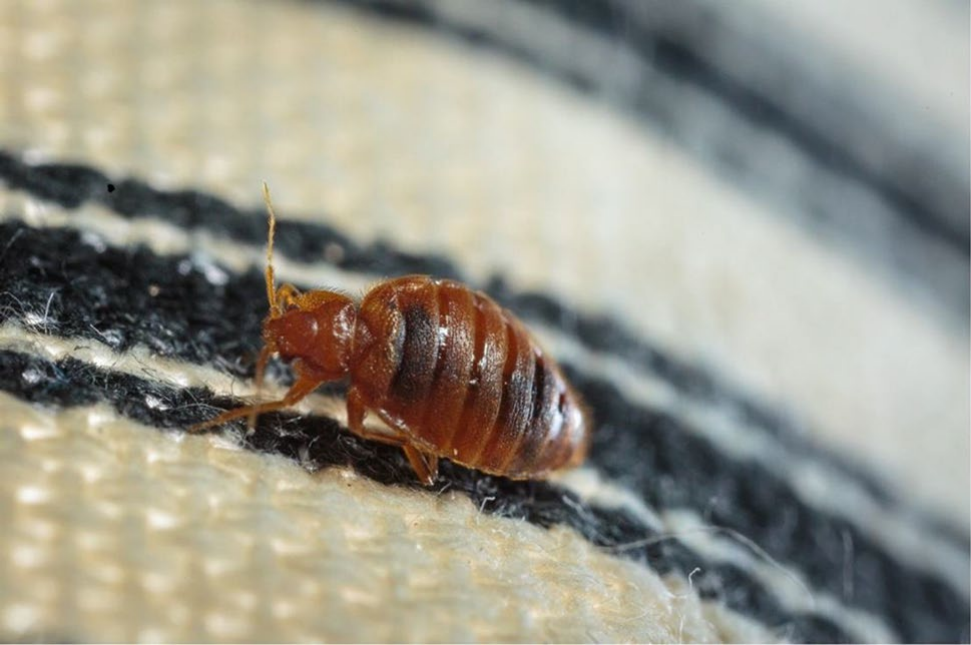
Bed bug. Bed bug (*Cimex lectularius*) on upholstery. Photo Credit: Matthew Barton, University of Kentucky.

The common bed bug (*Cimex lectularius* L.) (Fig. 1) is a blood-feeding indoor pest in the order Hemiptera. Infestations can grow to large sizes within homes, thus, their potential impacts on human health are of concern. Though their ability to transmit disease has not been confirmed outside of laboratory studies^1,2^, sensitivity to bed bug saliva has been identified^3,4^. Price et al. found Immunoglobulin E (IgE) response following cutaneous exposure to a ground up bed bug slurry in many patients, as well as indications of cross-reactivity between bed bug extracts and American house dust mite (*Dermataphagoides farina*) and German cockroach allergens^4^. While this study demonstrates IgE response against bed bug extract in study participants, specific allergens that might be responsible for patient reactions as well as cross-reactivity with extracts from other indoor arthropods were not identified.

Tropomyosin, along with other regulatory proteins, drives muscle contraction and relaxation throughout Eukaryota^5^. As a highly conserved structural protein in invertebrates, tropomyosin is likely present in most, if not all, invertebrates, including all insects. Invertebrate tropomyosin’s unique structure leads to sensitivity and allergic response in humans that is not documented with exposure to vertebrate tropomyosin^6^. Allergic responses to tropomyosin from indoor arthropods such as cockroaches^7–9^ and house dust mites^10^ have been identified, and cross-reactivity has been observed between shrimp and chironomid midges^11^ and orthopterans^12^, which further supports the broad reach of tropomyosin as an allergen across all invertebrate taxa. The official allergen database maintained by the World Health Organization and International Union of Immunological Societies (WHO/IUIS) Allergen Nomenclature Sub-Committee currently lists 40 allergenic tropomyosins from arthropod including crustaceans, mollusks, arachnids (house dust mite), and insects (http://www.allergen.org). Cross-reactivity occurs when an antibody raised against one allergen successfully binds to an allergenic protein from a different source, resulting in a comparable immune response^13^. The high degrees of structural homology between the forms of tropomyosin found across invertebrate taxa explains cross-reactivity between distantly-related species and establishes tropomyosin as a pan-allergen^6,14^.

The potential for sensitization to one or numerous invertebrates resulting in negative health impacts, such as anaphylaxis, from an entirely different species to which individuals were not previous sensitized to and through different routes of exposure (e.g., ingestion of tropomyosin-containing foods versus inhalation) have been highlighted across the literature^14–17^. Occupational asthma associated with crustaceans has been identified in several studies^18,19^. Cross-reactivity between tropomyosin allergens in shrimp and scallops was identified by Goetz and Whisman^20^, in the form of both dermal and inhalant responses across both of these taxa in a seafood handler. Additionally, crustacean tropomyosin has been implicated as an aerosolized allergen within seafood processing facilities, where muscle proteins can be broken into respirable particles through the cleaning, maceration, and steaming (cooking) of crabs^21–25^, scallops^20,26^ and squid^27^. Thus, there is a critical need to identify other potential sources of tropomyosin, especially from arthropods that people may have substantial exposure to.

Bed bugs as a potential source of respirable allergen has gained traction following the discovery of high levels of histamine in bed bug infested homes^28,29^. This finding is particularly concerning given the large quantities of histamine bed bugs can excrete^30^ and the unique ability of bed bugs to contaminate the indoor environment with histamine^31^. Histamine, a critical modulator within the mammalian system, can lead to significant health effects on humans through consumption^32^, dermal contact^33^, and respiration^34,35^. Given their close proximity to where we sleep and thus spend a majority of our time^36^, there is a need to investigate other possible inhalant allergens produced by bed bugs. It has been speculated that bed bugs produce inhalant allergens other than histamine, and the need for further study of bed bug allergens has been highlighted within the literature^9^.

Following a global resurgence in the late 1990s and early 2000s^37^, efforts have been made to identify effective bed bug control strategies^37^. Given the cryptic nature of bed bugs along with widespread pyrethroid resistance, eliminating bed bugs from homes is challenging. Two common methods for bed bug control that have emerged have been heat treatments and desiccant dusts (e.g., silica dust)^37^. Heat treatments involve heating the space to ensure consistent temperatures of at least 50 °C throughout the structure, which is effective at killing all life stages, including eggs^38^. While effective, care must be taken to address heat refugia that can insulate bed bugs against lethal temperature, which can lead to re-infestation^39^. Silica dust is one of few methods that provide effective residual control and that are effective against pyrethroid-resistant bed bugs^40^. It is often applied as a perimeter application and as crack and crevice treatment^41^.

The present study compared bed bug tropomyosin isoforms with other known tropomyosin allergens from other arthropod taxa to gain insights into potential allergenicity of bed bug tropomyosin, followed by an examination of bed bug bodies and associated products (e.g., feces and exuviae) for tropomyosin. Furthermore, the persistence of tropomyosin in bed bugs over time and with exposure to common bed bug treatment methods was explored. Finally, this study surveyed pest management professionals to determine how common treatment practices might lead to tropomyosin exposure in bed bug infested homes. This information is discussed in relation to bed bug management and efforts to reduce human exposure to allergens.

## Materials and methods

### Study insects

All bed bugs used in this study were males from the Harold Harlan population, a known insecticide-susceptible population originally collected from Fort Dix, New Jersey, USA in 1973. Bed bug colonies were reared under standard laboratory conditions: 25 °C, 50% RH, and a photoperiod of 12:12 h (L:D) cycle. They were reared on folded cardstock harborage in plastic containers (size: 168 ml; Consolidated Plastics, Stow, Ohio, USA) and maintained on weekly feedings of human blood containing the anticoagulant citrate phosphate dextrose (CPD) solution (Kentucky Blood Center, Lexington, Kentucky, USA) using an artificial water bath feeding system^42^. Briefly, a water bath maintained at a temperature of 37 °C and water was circulated through a double layer jacketed beaker, inside of which blood was placed and contained using a thin membrane (perforated budding tape, 25 mm, A.M. Leonard, Piqua, Ohio, USA). This maintained blood temperature while bed bugs fed. All insects were starved for 1 week prior to use in experiments.

### Comparison of tropomyosin isoforms in bed bugs to other allergenic arthropod taxa

Tropomyosin isoforms identified from the common bed bug by Benoit et al.^43^ were determined using the National Centre for Biotechnology Information Blast (protein) database and compared with select tropomyosins of identified allergenic sources, such as those highlighted by Shafique et al.^44^ and Wong et al.^45^. Protein Blast was used to align and compare each bed bug tropomyosin isoform with known tropomyosin allergens from other invertebrates. Those bed bug amino acid sequences with ≥ 70% sequence identity to tropomyosin from identified allergenic taxa were considered strong candidates for cross-reactivity with the known tropomyosin allergens^46^. Furthermore, a percentage identity matrix was constructed between a representative bed bug tropomyosin isoform (X8, XP_014251831.1) with the highest sequence homologies to tropomyosins from other taxa and the 40 allergenic tropomyosins identified in the http://www.allergen.org data base as of November 2023 using Clustal Omega^47^. Only one isoallergen per tropomyosin within the database was used to construct the matrix. Microsoft Excel (version 15.78.3) was used to create the percent matrix identity from Clustal omega outputs.

### Characterization of tropomyosin from bed bug feces, exuviae, and bodies

To determine if tropomyosin can be detected from bed bug feces, a single male Harlan bed bug was provided a bloodmeal and allowed to defecate on a 1 × 1.5 cm cardstock rectangle (Office Depot 3-tab filing folders, #NF810838, Smead Manufacturing Company, Hastings, Minnesota, USA) for 1 week before the bed bug was removed and the feces-stained filter paper was extracted (*n* = 6). To determine if tropomyosin could be detected from bed bug exuviae, groups (*n* = 9) of 100 fourth/fifth instar exuviae were collected from Harlan bed bug colonies. The sample extraction procedure was modified from an InBio dust sample extraction procedure. Exuviae and feces samples were extracted in phosphate-buffed saline (PBS) with Tween 20 (0.05%; PBS-T) by mixing on a nutating rocker for 2 h. Supernatants were centrifuged for 3 min at 437*g*, removed and transferred to a clean vial and stored at – 20 °C until analysis.

To characterize tropomyosin from whole bed bug bodies (*n* = 10), male bed bugs were killed by freezing at − 20 °C for 24 h before extraction in PBS-T using the above method. To simulate environmental conditions in homes where dead bed bugs may be fragmented or ground up by moving furniture or being stepped on, bed bugs were fragmented (*n* = 9) and compared to unfragmented dead bed bugs. To achieve fragmentation, male Harlan bed bugs killed by freezing were placed in a Fisher Scientific Isotemp Incubator model 655D (Fischer Scientific, Pittsburgh, Pennsylvania, USA) set to 26 °C and 15% RH for 44 h to remove moisture. Then, individual dried bed bugs were ground up manually using the tip of a glass pipette to break the body into fragments of ~ 1 mm or less, before being extracted in PBS-T.

Analysis of samples was conducted using an enzyme-linked immunosorbent assay (ELISA) specific for tropomyosin (InBio, Charlottesville, Virginia, USA). Briefly, each well of a 96-well microplate (EIA/RIA Clear Flat Bottom Polystyrene High Bind Microplate, Corning^®^ Life Sciences, Corning, New York, USA) was coated with 100 µl anti-tropomyosin primary antibody (MA-1A6, 1 mg/ml in phosphate-buffered saline (PBS), pH 7.4, InBio, Charlottesville, Virginia, USA) diluted 1:1000 in PBS. After incubation at 4 °C overnight, the antibody was discarded, and wells were blocked with 100 µl of 1% bovine serum albumin (BSA) in PBS-T (A7030, Sigma-Aldrich Inc., St. Louis, Missouri, USA) for 30 min. All subsequent incubation times were for 1 h and plates were triple-rinsed with PBS-T between steps. Twenty µl of purified natural shrimp tropomyosin prepared in 1% BSA/50% glycerol/PBS at a concentration of 5000 ng/ml (NA-STM-1, InBio, Charlottesville, Virginia, USA) was added to 180 µl of BSA PBS-T in corresponding wells for generation of the standard curve, which consisted of twofold dilutions from 500 to 0.98 ng/ml. Additionally, 20 µl of sample supernatant was added to 180 µL BSA PBS-T in corresponding wells for each sample, then run in twofold serial dilutions across four wells per sample. Blanks were 100 µl of BSA PBS-T. One hundred µl of rabbit anti- shrimp tropomyosin polyclonal antibody (PA-SHM, InBio, Charlottesville, Virginia, USA) diluted 1:1000 in 1% BSA PBS-T were then added to each well, followed by incubation with 100 µl of peroxidase-conjugated goat anti-rabbit immunoglobulin diluted 1:1000 in 1% BSA PBS-T (Jackson Immunoresearch Laboratories, West Grove, Pennsylvania, USA). After the final incubation and rinse, 100 µl of 1mM ABTS in 70 mM citrate–phosphate buffer (Diammonium 2,2′-azinobis[3-ethyl-2,3-dihydrobenzothiazole-6-sulfonate, Sigma Aldrich, St. Louis, Missouri, USA) containing 0.03% hydrogen peroxide (30% w/w, Honeywell, Muskegon, MI) were added to all wells and plates were read with a microplate spectrophotometer (Biotek^®^ Powerwave X, Winooski, Vermont, USA) at a wavelength of 405 nm. The shrimp tropomyosin standard was used to create a four-point logistic standard curve. Study data were fit to the curve to calculate tropomyosin concentrations in each sample. All values calculated below the standard curve were given a value of 0.

### Characterization of tropomyosin in bed bug bodies with age

To evaluate amount of tropomyosin in bed bug cadavers with aging, bed bugs were killed by freezing at – 20 °C for 24 h. Intact bed bug cadavers were removed from freezer and aged for 1 month, 3 months, 6 months, and 18 months at ambient relative humidity and temperature (22–23 °C, 45–55% RH) before being fragmented and extracted in PBS-T. Ten replicates were performed for each of the four aging time points.

### Characterization of tropomyosin in silica dust- and heat-killed bed bugs

To determine if tropomyosin could be detected from bed bugs killed via a standard pesticide application, 1 week starved male bed bugs (*n* = 10) from the Harlan population were exposed to a plastic arena (35.6 × 20.3 × 12.4 cm, Sterlite, Davenport, Iowa, USA ) where silica dust (CimeXa^®^, Rockwell Laboratories, Kansas City, Missouri, USA) had been applied as a “light, visible film” at an application rate of 2oz/100 sq feet (~ 0.0061 kg/m^2^) using a handheld pesticide duster (Southern Homewares P/N 818947013256, Fairly Odd Treasures, LLC., Charlotte, North Carolina, USA) per label instructions. Mortality was confirmed after 24 h continuous exposure and individual bed bugs were fragmented and extracted in PBS-T. To examine tropomyosin following exposure to heat treatment, live 1 week starved males (*n* = 10) from Harlan colonies were placed in a Fisher Scientific Isotemp Incubator model 655D for 90 min at ~ 50 °C and 15% RH with no harborage^37,38^. Once removed from the incubator, mortality was confirmed and bed bugs were fragmented and extracted following the above protocol and analyzed using ELISA.

### Questionnaire of the pest control industry on bed bug management protocols

An electronic questionnaire was distributed to members of the pest control industry via emails by industry publications: Pest Control Technology (GIE Media, Inc., 5811 Canal Road, Valley View, OH 44125) and Pest Management Professional (North Coast Media LLC., 1360 East 9th St., 10th Floor, Cleveland, OH 44114). Approval of the survey was obtained from the Institutional Review Board (IRB) of the University of Kentucky (Protocol # 8746) and survey data was collected in accordance with relevant guidelines/regulations, including informed consent of all participants. Questionnaire data collected was anonymous and included: position within the industry, treatments used by their company for bed bug treatments, and if treatment protocols include removal of live bed bugs or dead bed bugs following treatment. Survey recruitment began on May 26th, 2023 and concluded on July 13th, 2023.

### Statistical analyses

All statistical analyses were done in R version 3.6.2^48^. Analysis of variance (ANOVA) was used to assess the impact of age on detectable tropomyosin from fragmented bed bug cadavers, with Tukey’s post hoc testing used to assess pairwise differences. Mann–Whitney U Test was used to compare mean tropomyosin from whole and fragmented bed bugs.

## Results

### Comparison of tropomyosin isoforms in bed bugs to other taxonomic groups

Twenty three total isoforms of tropomyosin have been identified from the common bed bug^43^ (Table 1). Of these, all (23/23) isoforms shared ≥ 70% identities with German cockroach (*Blattella germanica*) tropomyosin and American cockroach (*Periplaneta americana*) tropomyosin, followed by 22/23 isoforms compared with northern brown shrimp (*Farfantepenaeus aztecus*), 22/23 isoforms compared with crucifix crab (*Charybdis feriata*), 22/23 bed bug isoforms compared with American lobster (*Homarus americanus*), 19/23 isoforms compared with American house dust mite (*Dermatophagoides farinae*), and 18/23 isoforms compared with European house dust mite (*Dermatophagoides pteronyssinus*) (Table 2).

**Table 1:** Tropomyosin isoforms identified from the genome of the common bed bugs (*Cimex lectularius*) by Benoit et al.^43^.

*Cimex lectularius* tropomyosin isoforms	NCBI reference sequence
Tropomyosin-2 isoform X1	XP_014251790.1
Tropomyosin isoform X2	XP_024082820.1
Tropomyosin isoform X3	XP_024082821.1
Tropomyosin isoform X4	XP_014251800.1
Tropomyosin isoform X5	XP_014251810.1
Tropomyosin isoform X6	XP_024082822.1
Tropomyosin isoforms c/e isoform X7	XP_014251819.1
Tropomyosin isoform X8	XP_014251831.1
Tropomyosin isoform X9	XP_014251839.1
Tropomyosin isoform X10	XP_024082823.1
Tropomyosin isoform X11	XP_014251844.1
Tropomyosin isoform X12	XP_014251851.1
Tropomyosin isoform X13	XP_024082824.1
Tropomyosin isoform X14	XP_014251857.1
Tropomyosin isoform X15	XP_014251870.1
Tropomyosin isoform X16	XP_014251880.1
tropomyosin-1, isoforms 9A/A/B isoform X17	XP_024082825.1
Tropomyosin-1, isoforms 9A/A/B isoform X18	XP_024082826.1
Tropomyosin-1, isoforms 9A/A/B isoform X19	XP_014251889.1
Tropomyosin-1, isoforms 9A/A/B isoform X20	XP_024082827.1
Tropomyosin-1, isoforms 9A/A/B isoform X21	XP_014251897.1
Tropomyosin-1, isoforms 9A/A/B isoform X22	XP_014251922.1
Tropomyosin-1, isoforms 9A/A/B isoform X23	XP_014251913.1

**Table 2:** Comparison of tropomyosin allergens from other select invertebrates with tropomyosin isoforms identified from the common bed bug (*Cimex lectularius*) by Benoit et al.^43^.

Tropomyosin isoforms from the common bed bug *(Cimex lectularius*)	German cockroach	American cockroach	European house dust mite	American house dust mite	Northern brown shrimp	Crucifix crab	American lobster
	*Blattella germanica*	*Periplaneta americana*	*Dermatophagoides pteronyssinus*	*Dermataphagoides farina*	*Farfantepenaeus aztecus*	*Charybdis feriata*	*Homarus americanus*
	Bla g 7	Per a 7.0101	Der p 10	Der f 10	Pen a 1	Cha f 1	Hom a 1
	AAF72534^a^	CAB38086.1	CAA75141^a^	BAA04557^a^	AAZ76743^a^	AAF35431^a^	AAC48288^a^
X1	**175/204 (86%)**	**176/204 (86%)**	**156/204 (76%)**	**159/204 (78%)**	**174/204 (85%)**	**168/187 (90%)**	**173/204 (85%)**
X2	**169/204 (83%)**	**169/204 (83%)**	**150/204 (74%)**	**153/204 (75%)**	**168/204 (82%)**	**161/187 (86%)**	**181/204 (88%)**
X3	**159/204 (78%)**	**160/204 (78%)**	**145/204 (71%)**	**147/204 (72%)**	**158/204 (77%)**	**152/187 (81%)**	**157/204 (77%)**
X4	**168/204 (82%)**	**168/204 (82%)**	**155/204 (76%)**	**157/204 (77%)**	**168/204 (82%)**	**163/187 (87%)**	**168/204 (82%)**
X5	**153/204 (75%)**	**153/204 (75%)**	139/204 (68%)	141/204 (69%)	**152/204 (75%)**	**145/187 (78%)**	**151/204 (74%)**
X6	**162/204 (79%)**	**161/204 (79%)**	**149/204 (73%)**	**151/204 (74%)**	**162/204 (79%)**	**156/187 (83%)**	**162/204 (79%)**
X7	**146/204 (72%)**	**145/204 (71%)**	138/204 (68%)	139/204 (68%)	**146/204 (72%)**	**140/187 (75%)**	**146/204 (72%)**
X8	**189/206 (92%)**	**189/206 (92%)**	**166/206 (81%)**	**169/206 (82%)**	**184/206 (89%)**	**169/187 (90%)**	**183/206 (89%)**
X9	**182/206 (88%)**	**174/206 (84%)**	**165/206 (80%)**	**167/206 (81%)**	**178/206 (86%)**	**164/187 (88%)**	**178/206 (86%)**
X10	**176/206 (85%)**	**158/206 (77%)**	**159/206 (77%)**	**161/206 (78%)**	**172/206 (83%)**	**157/187 (84%)**	**172/206 (83%)**
X11	**160/206 (78%)**	**158/206 (77%)**	**148/206 (72%)**	**149/206 (72%)**	**156/206 (76%)**	**141/187 (75%)**	**156/206 (76%)**
X12	**245/281 (87%)**	**246/281 (88%)**	**217/284 (76%)**	**221/284 (78%)**	**229/284 (81%)**	**227/264 (86%)**	**229/284 (88%)**
X13	**229/281 (81%)**	**230/281 (82%)**	**206/281 (73%)**	**209/281 (74%)**	**213/284 (75%**	**211/264 (80%)**	**213/284 (75%)**
X14	**223/281 (79%)**	**223/281 (79%)**	**200/284 (70%)**	**203/284 (71%)**	**207/284 (73%)**	**204/264 (77%)**	**207/284 (73%)**
X15	**259/283 (92%)**	**259/283 (92%)**	**227/283 (80%)**	**231/283 (82%)**	**239/283 (84%)**	**228/264 (86%)**	**239/283 (84%)**
X16	**66/93 (71%)**	**65/93 (70%)**	58/93 (62%)	58/93 (62%)	64/93 (69%)	63/93 (68%)	64/93 (69%)
X17	**168/201 (84%)**	**168/201 (84%)**	**149/203 (73%)**	**152/203 (75%)**	**167/204 (82%)**	**160/184 (87%)**	**166/204 (88%)**
X18	**158/201 (79%)**	**159/201 (79%)**	**144/201 (72%)**	**146/201 (73%)**	**157/201 (78%)**	**151/184 (82%)**	**156/201 (78%)**
X19	**152/201 (76%)**	**165/203 (81%)**	138/201 (69%)	**140/201 (70%)**	**151/204 (74%)**	**144/184 (78%)**	**150/204 (74%)**
X20	**161/201 (80%)**	**160/201 (80%)**	**148/204 (73%)**	**150/204 (74%)**	**161/204 (79%)**	**155/184 (84%)**	**161/204 (79%)**
X21	**145/201 (72%)**	**144/201 (72%)**	137/201 (68%)	138/201 (69%)	**145/204 (71%)**	**139/184 (76%)**	**145/204 (71%)**
X22	**172/203 (85%)**	**172/203 (85%)**	**154/203 (76%)**	**156/203 (77%)**	**167/203 (82%)**	**152/184 (83%)**	**166/203 (82%)**
X23	**166/203 (82%)**	**165/203 (81%)**	**148/203 (73%)**	**150/203 (74%)**	**161/203 (79%)**	**145/184 (79%)**	**160/203 (79%)**

Sequences with > 70% homology are in bold.

^a^Genbank Accession number.

In creation of a percentage identity matrix using bed bug tropomyosin X8 as a representative isoform for bed bug tropomyosin (Fig S1), several taxa from the WHO/IUIS Allergen Nomenclature Sub-Committee database were necessarily excluded due to lack of GenBank protein sequence: *Bombyx mori*, *Exopalaemon modestus*, *Helix aspersa*, *Penaeus indicus*, *Saccostrea glomerata*, *Todarodes pacificus*. Across the 33 allergenic taxa, bed bug tropomyosin X8 displayed between 43 and 72% similarities, with the highest similarities to *B. germanica* (72.34%) and *P*. *americana* (72.70%). Bed bug tropomyosin X8 had the lowest similarities to the iridescent shark catfish, *Pangasianodon hypophthalmus* (43.97%) and the Atlantic salmon, *Salmo salar* (46.45%).

### Characterization of tropomyosin from bed bug feces, exuviae, and bodies

**Figure 2:**
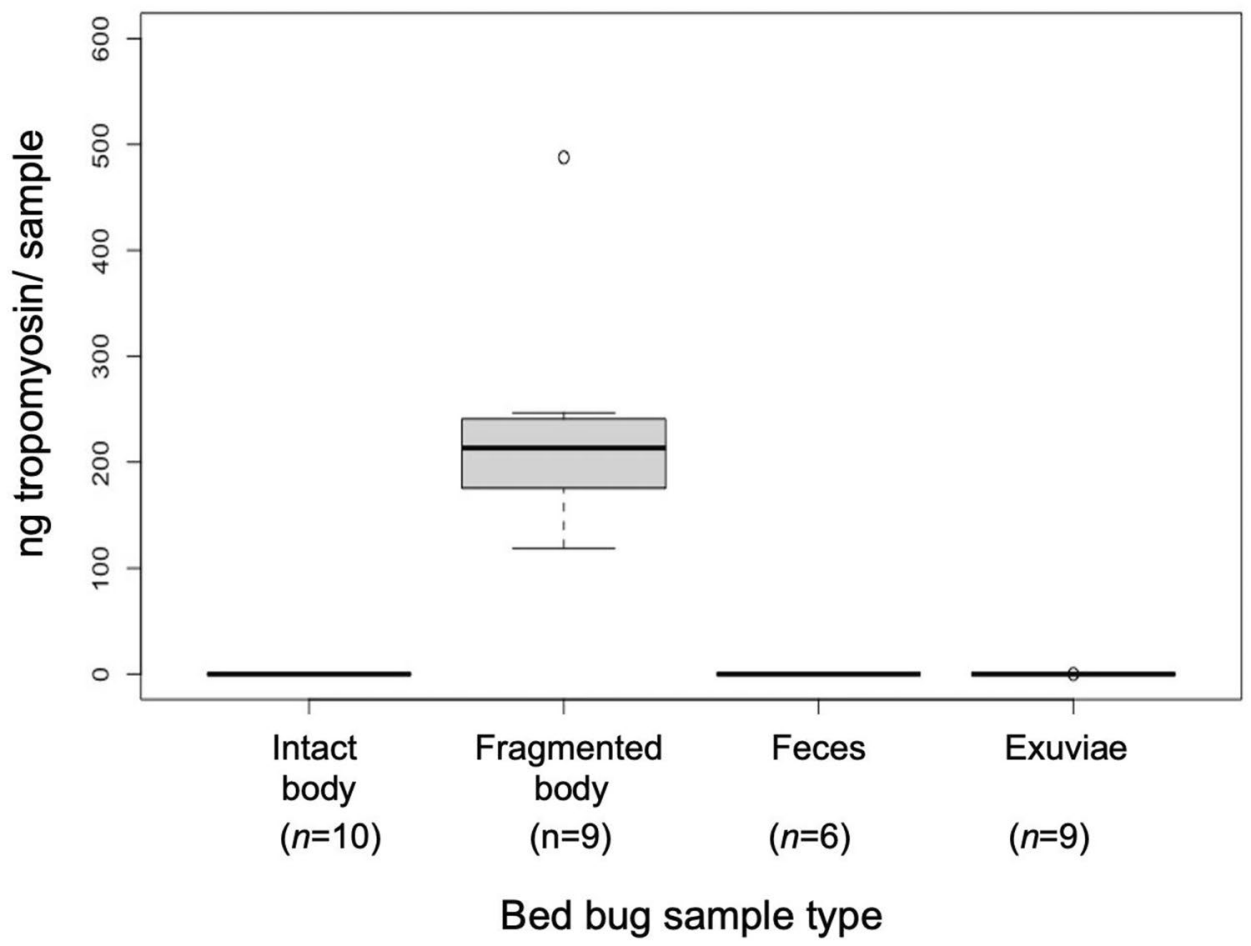
Tropomyosin from bed bug products. Comparisons of mean ng tropomyosin detected in samples of singular intact or fragmented bed bug bodies, 1 week of fecal deposits for a single bed bug, and 100 bed bug exuviae (cast skins).

No tropomyosin was detected from any of the feces samples (Fig. 2). Very small amounts of tropomyosin were detected in exuviae, with an average of 0.54 ± 0.00 ng/100 exuviae detected in samples with detectable tropomyosin (Fig. 2). In 4 out of 9 exuviae samples, no tropomyosin could be detected at all. All whole bed bug samples were calculated below the standard curve and were given a value of 0 and an average of 223.44 ± 36.29 ng/bed bug could be quantified from fragmented bed bugs (Fig. 2). Fragmented bed bug bodies had significantly greater tropomyosin than whole bed bug (bodies *U* = 0, p < 0.0001).

### Characterization of tropomyosin in bed bug bodies with age

**Figure 3:**
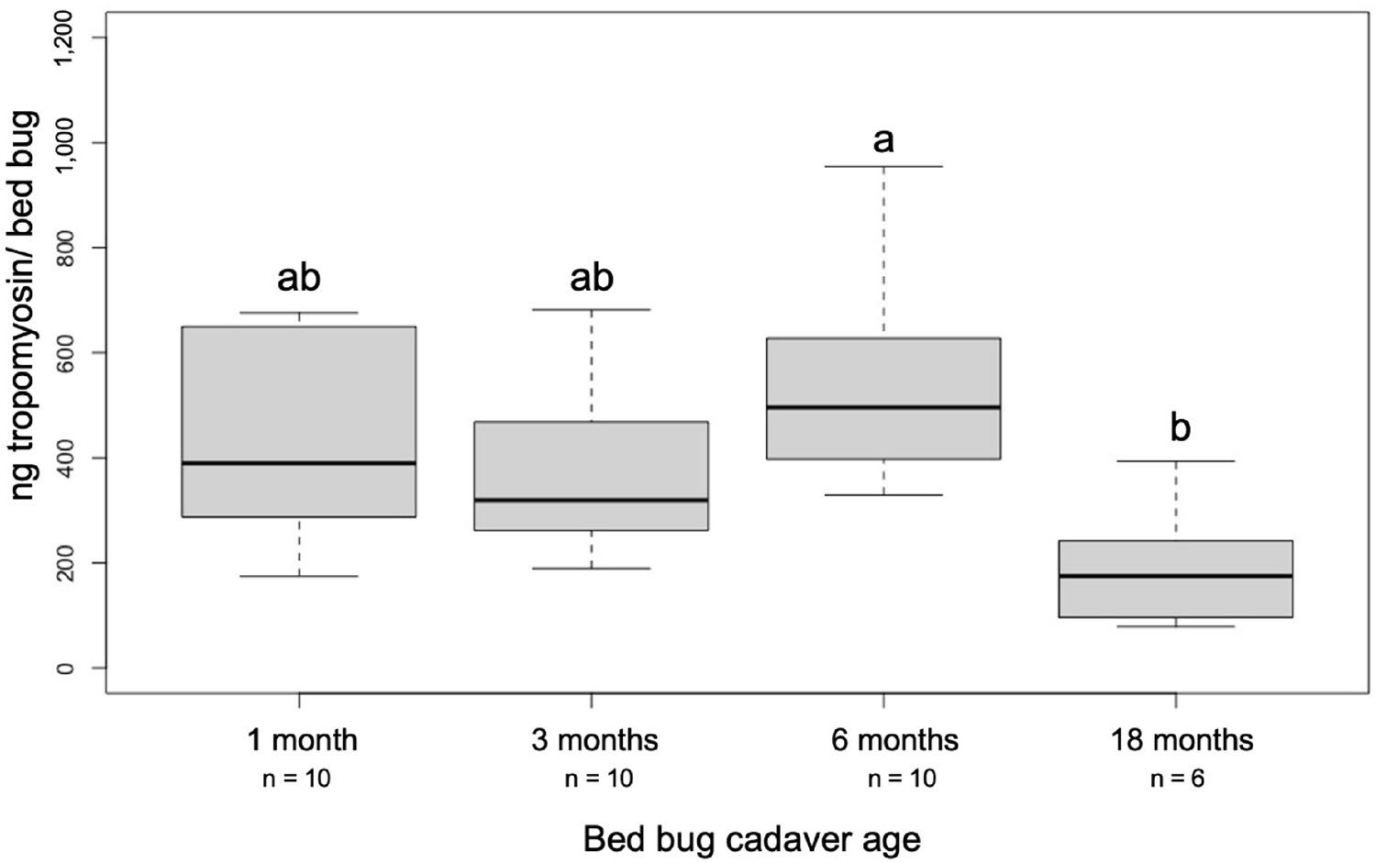
Bed bug tropomyosin with aging. Comparisons of mean ng tropomyosin/bed bug detected after different time points from samples of mechanically fragmented bed bug cadavers. Different letters indicate significant differences between group means based on Tukey’s post hoc testing.

Aging had a significant impact on the amount of tropomyosin detected from bed bug bodies upon fragmentation (*F*(3, 32) = 5.812, *p* = 0.003) (Fig. 3). Six months of aging prior to fragmentation had the highest average tropomyosin detected (551.90 ± 60.75 ng/bed bug), though a Tukey post-hoc test found no significant difference between 1, 3 and 6 months. There was a significant decrease in tropomyosin between 6 and 18 months, though the average tropomyosin that could be detected from bed bug bodies that had aged for 18 months before fragmentation did not differ from 1 and 3 months of aging.

### Characterization of tropomyosin in silica dust- and heat-killed bed bugs

An average of 62.08 ± 8.25 ng tropomyosin/bed bug was quantified from silica-dust-killed bed bugs and an average of 329.31 ± 13.79 ng/bed bug was quantified from heat-killed bed bugs.

### Questionnaire of the pest control industry on bed bug management protocols

Of the 63 responses received, 8 were excluded due to incomplete responses. 55 responses remained and were used for further analysis (Table 3). Of these responses, seven indicated they were technicians, 11 managers, 12 in technical services, two in sales, 17 owners, and six indicated ‘other’. Of those that marked ‘other’, roles included president, researcher, COO, field training director, and university staff. 22% (12/55) indicated they only use one of the treatment strategies for bed bugs (heat, chemical, or biopesticide), while 78% (43/55) selected more than open choice. Of those that selected one treatment, chemical was the most selected, followed by biopesticides, heat, and other, respectively. Out of 51 responses for questions relating to vacuuming as a part of treatment plans, 82% (42/51) of respondents stated that their company vacuums/removes live bed bugs as part of the treatment protocol. When asked if their company vacuums/removes dead bed bugs following treatment, only 39% (20/51) selected ‘yes’.

**Table 3:** Treatment and bed bug removal practices by pest management professionals.

What treatments does your company use for bed bugs? Please select all that apply (55)	Heat	Chemical	Biopesticide	Other
	24 (44%)	42 (76%)	29 (53%)	15 (27%)
Does your treatment protocol for bed bugs include vacuuming and removal of live bed bugs? (53)		Yes	No	Unsure
		43 (81%)	10 (19%)	0 (0%)
Does your treatment protocol for bed bugs include cleaning up/removal of dead bed bugs following treatment? (51)		Yes	No	Unsure
		20 (39%)	31 (61%)	0 (0%)

## Discussion

Our findings provide a baseline that confirms bed bugs contain tropomyosin, a known allergen within other invertebrate taxa. Given the clear homologies between bed bug tropomyosin and tropomyosin allergens from other arthropod pests within the home (cockroaches and house dust mite), and between bed bug tropomyosin and tropomyosin from shrimp and lobster, bed bugs represent an additional and significant source of tropomyosin within the home that is likely capable of causing an allergic reaction. While this study speculates on the allergenicity of bed bug tropomyosin due to the sequence homology of multiple isoforms of bed bug tropomyosin to tropomyosin from known allergenic taxa, additional studies are needed to confirm the allergenicity of bed bug tropomyosin.

No tropomyosin was identified from feces and only small quantities of tropomyosin were detected from half of the exuviae samples. Tropomyosin has been identified from the gut of other members of Hemiptera^49^ and it is possible small amounts of tropomyosin may have become attached to the chitinous lining of the alimentary canal, which would explain the trace amounts of tropomyosin detected from exuviae. More likely, since exuviae were collected directly from colony rearing jars, where groups of hundreds to thousands of bed bugs are growing and molting together, small quantities of tropomyosin identified from some of the exuviae samples in this study could be due to tropomyosin contamination from dead bed bugs within the jars. If nymphal bed bugs had been isolated and allowed to molt, we suspect this clean exuviae would not contain detectable tropomyosin.

In our study, no tropomyosin could be detected from freshly-killed, intact bed bugs. In contrast, 223 ng of tropomyosin per bed bug could be found from freshly-killed and ground up bed bugs, indicating bed bug bodies need to be broken to release tropomyosin into the indoor environment. In large infestations, bed bugs can number in the tens of thousands^37^, which can amount to a substantial amount of tropomyosin entering the indoor environment. Infestations include adult females as well as immature life stages. Due to its function as a muscle protein, it is probable that tropomyosin is present in both sexes and all life stages, though there may be differences based on individual size (i.e., a first instar nymph would contain less tropomyosin than a mature adult). While this study represented an initial exploration into tropomyosin from bed bugs, additional studies should evaluate these differences, as well as any potential differences between insecticide-susceptible laboratory populations (tested here) compared with insecticide-resistant field-collected populations. The increases in tropomyosin with aging from 3 to 6 months of bed bug cadavers prior to grinding may be due to the breakdown of tissues following death. Decomposition might have released more of the protein previously bound up in the muscle tissue, which could then bind to capture antibodies during ELISA. Additionally, the mechanical breakdown of bed bug bodies might have been more effective once the bed bug cadaver had dried. Impacts of decomposition and enzymes responsible for tissue degradation on tropomyosin release into the home environment should be examined. The amount of tropomyosin that could be quantified from bed bug bodies appears to decline after prolonged aging, given the lower tropomyosin quantified from 18-month-aged samples compared to 6-month-aged. However, the quantity of tropomyosin after 18 months of aging was not significantly lower than 1 or 3 months, suggesting that this decline in tropomyosin with the aging of bed bug bodies is a relatively slow process.

Tropomyosin could still be detected from heat-killed and silica dust-killed bed bugs, with an average of 329.3ng/bed bug and 62.1 ng/bed bug, respectively. Our findings confirm the heat-stability of tropomyosin found in other taxa, hypothesized to be due to its structure^6^. However, we did not see increased monoclonal antibody binding with exposure to heat, as found in other studies^50,51^. Kamath et al.^50^ heated the proteins to 100 °C and in boiling PBS, which are substantially higher temperature than the 50 °C recommended for bed bug treatment^38^ and used in the present study. It is possible that bed bug tropomyosin may undergo similar increases in detection and reactivity if heated to similar temperatures as in studies dealing with crustacean tropomyosin. A lower amount of tropomyosin was quantified from bed bugs killed with silica dust than with heat. Silica particles are used within chromatography columns to bind and separate proteins for purification and analysis^52^. Adhesion of proteins to silica dust particles could be responsible for their lack of binding, and therefore detection, in our ELISA. Further work should investigate the potential for silica dust binding to tropomyosin proteins and its implications for tropomyosin availability and impacts on human health within respirable dust. Additionally, desiccant dusts work on bed bugs by absorbing the outer wax layer of the insect’s cuticle, resulting in moisture loss and eventual death^53,54^. In the present study, the desiccation resulting from silica dust exposure may have been sufficient to cause mortality and allow for fragmenting of the bed bug body, but it is possible that the degree of dryness was less than when insect bodies were placed in the drying oven (fresh bodies, fragmented), aged at room temperature for longer time points (bodies aged for 1+ months), and subjected to high temperatures to mimic a bed bug heat treatment. This lower degree of desiccation in silica-dust-killed bed bugs may have resulted in less tropomyosin exposed and available for quantification. However, the impacts of both silica dust and heat treatments on desiccation of the insect body can likely expedite the process for bed bug bodies to become brittle enough to release the maximum quantity of tropomyosin such as that seen after 6 months of aging under ambient conditions.

Our findings demonstrate that the dead remains of bed bugs (such as what would remain in the home following treatment) can not only contain tropomyosin, but potentially continue to introduce this allergen into the home as the remains break down naturally or through unintended breaking such as being stepped on or crushed through the moving of furniture, or even by vacuuming without a HEPA filter. We also found that tropomyosin is unaffected by traditional bed bug treatments involving heat and silica dust applications. Out of the 51 responses to our survey of pest management professionals, only 20 (39%) stated that “removal of dead bed bugs following treatment” was part of their standard operating procedures. To reduce continued exposure of residents to tropomyosin, it may be necessary to remove dead bed bugs after treatments, although this has not been tested. The longevity of tropomyosin in the indoor home environment, as well as impacts of aging on IgE-reactivity to tropomyosin in sensitized individuals should also be explored.

The case for cross-reactivity of tropomyosin across arthropod groups has been extensively made^6,14,44^ and the cross-reactivity between cockroaches and house dust mites^44,45^ likely extends to bed bugs as well. Exposure and subsequent sensitization to bed bug tropomyosin may contribute to the “covariation of sensitization” seen across other arthropod groups^44^ and individuals sensitized to tropomyosin from house dust mite and/or cockroaches may experience higher reactivity to tropomyosin from bed bugs and vice versa. Cross-sensitization between bed bugs and other indoor arthropods such as cockroaches and house dust mites has the potential to increase risks of health impacts for residents in homes with chronic bed bug and/or cockroach infestations. Pest control operators that are working in multiple homes, and often coming into contact with multi-pest infestations, may also have increased risk of health impacts resulting from this cross-reactivity. If the allergenicity of bed bug tropomyosin is confirmed, it would not only translate to potential development of allergic responses to bed bugs, but potential development and exacerbation of responses to cockroaches and house dust mite as well. Furthermore, due to the homologies of a majority of bed bug tropomyosin isoforms to tropomyosin allergens from lobster, crab, and shrimp (Table 2), exposure to indoor arthropod tropomyosin may lead to the development of immune response to consumed tropomyosin in shellfish.

Future studies should screen human subjects for both IgE hypersensitivity to bed bug tropomyosin and IgG sensitivity in individuals with sensitivity to other tropomyosin allergens, such as those from shrimp or cockroaches, but who have not had previous exposure to bed bugs. Additionally, due to the presence of dead bed bug fragments becoming incorporated within household dust, future studies should explore if inhalant exposure to bed bug tropomyosin can lead to sensitization and symptoms such as allergic rhinitis and bronchial hyperreactivity.

### Supplementary Information

Supplementary Information.

## Data Availability

Bed bug tropomyosin data analyzed during this study are included in the supplementary materials of this article (Table S1).
