# Supplementary material for: Identification of the pan-allergen tropomyosin from the common bed bug (Cimex lectularius)
Source: Sci Rep. 2024 Mar 27;14:7281. doi: 10.1038/s41598-024-57877-3 (PMC10973433; doi:10.1038/s41598-024-57877-3)
Supplement: Supplementary file 1 — Supplementary Information. [file 41598_2024_57877_MOESM1_ESM.pdf]

	Pangasianodon hypophthalmus	*Oreochromis mossambicus*	*Salmo salar*	*Haliotis laevigata x Haliotis rubra*	*Crassostrea angulata*	*Crassostrea gigas*	*Lepisma saccharina*	*Cimex lectularius*	*Anisakis simplex*	*Coptotermes formosanus*	*Blatella germanica*	*Periplaneta americana*	*Aedes aegypti*	*Chironomus kiiensis*	*Dermatophagoides farinae*	*Dermatophagoides pteronyssinus*	*Lepidoglyphus destructor*	*Blomia tropicalis*	*Chortoglyphus arcuatus*	*Homarus americanus*	*Charybdis feriatus*	*Scylla paramamosain*	*Melicertus latisulcatus*	*Procambarus clarkii*	*Panulirus stimpsoni*	*Portunus pelagicus*	*Litopenaeus vannamei*	*Penaeus aztecus*	*Penaeus monodon*	*Metapenaeus ensis*	*Crangon crangon*	*Macrobrachium rosenbergii*	*Pandalus borealis*
Pangasianodon hypophthalmus	100.00	82.39	80.28	53.07	50.90	51.62	48.19	43.97	55.07	51.61	51.81	50.72	53.26	51.45	55.80	55.07	56.52	55.80	55.80	54.71	56.64	54.71	54.35	54.35	53.01	53.62	53.62	53.62	53.62	52.63	54.35	54.35	55.43
Oreochromis mossambicus	82.39	100.00	95.42	55.96	55.60	56.32	49.28	47.16	58.70	55.65	54.71	54.35	58.33	56.16	58.33	57.61	57.61	58.33	57.97	58.33	60.55	58.70	58.70	57.97	56.77	57.61	57.97	57.97	57.97	56.77	58.70	58.70	59.42
Salmo salar	80.28	95.42	100.00	55.23	55.23	55.96	49.64	46.45	57.97	55.24	54.35	53.99	57.61	55.80	58.33	57.61	57.97	58.70	58.33	57.61	59.77	57.97	57.61	57.61	56.39	57.25	57.61	57.61	57.61	56.39	57.61	57.61	58.33
Haliotis laevigata x Haliotis rubra	53.07	55.96	55.23	100.00	78.52	82.39	56.07	53.55	63.57	59.13	61.79	62.14	62.14	61.07	65.00	63.57	63.57	64.64	64.64	63.93	61.92	63.57	64.64	63.93	63.33	63.57	63.93	63.93	63.93	62.96	62.93	64.29	64.29
Crassostrea angulata	50.90	55.60	55.23	78.52	100.00	95.42	53.21	52.84	59.93	57.54	60.00	60.36	60.50	58.72	61.79	60.36	60.71	61.79	62.14	61.43	59.62	61.07	62.14	61.07	61.11	60.71	61.07	61.07	61.07	60.37	61.79	61.43	61.79
Crassostrea gigas	51.62	56.32	55.96	82.39	95.42	100.00	55.00	54.61	61.79	59.52	61.79	62.14	62.14	60.36	63.21	61.79	62.14	63.21	63.57	62.86	61.54	62.86	63.93	62.50	62.59	62.14	62.86	62.86	62.86	62.22	63.21	62.86	63.21
Lepisma saccharina	48.19	49.28	49.64	56.07	53.21	55.00	100.00	54.61	61.07	65.48	65.00	65.71	65.71	64.64	66.07	64.64	64.29	65.36	66.43	68.21	68.85	68.21	68.21	66.79	67.41	67.86	68.21	68.21	68.21	67.41	68.57	68.57	68.93
Cimex lectularius	**43.97**	**47.16**	**46.45**	**53.55**	**52.84**	**54.61**	**54.61**	**100.00**	**61.70**	**69.80**	**72.34**	**72.70**	**70.57**	**70.92**	**63.82**	**64.89**	**63.83**	**63.83**	**64.89**	**69.86**	**69.96**	**70.57**	**69.86**	**69.15**	**71.32**	**69.15**	**69.86**	**69.86**	**69.86**	**71.69**	**69.86**	**69.86**	**69.50**
Anisakis simplex	55.07	58.70	57.97	63.57	59.93	61.79	61.07	61.70	100.00	67.19	69.37	69.01	70.53	69.12	74.65	73.24	73.59	74.30	74.65	74.65	74.24	75.00	71.83	72.18	71.90	72.18	72.89	72.89	72.89	71.90	73.24	73.24	73.24
Coptotermes formosanus	51.61	55.65	55.24	59.13	57.54	59.52	65.48	69.80	67.19	100.00	96.48	96.88	90.23	87.50	79.30	78.52	76.95	76.95	78.12	81.25	82.42	82.81	80.47	80.08	80.49	81.25	81.25	81.25	81.25	80.49	81.64	80.86	81.25
Blatella germanica	51.81	54.71	54.35	61.79	60.00	61.79	65.00	72.34	69.37	96.48	100.00	97.18	89.44	86.97	80.63	79.58	78.52	78.52	79.58	83.10	84.09	83.10	82.04	82.04	82.12	82.04	82.39	82.39	82.39	81.75	82.75	82.04	82.39
Periplaneta americana	50.72	54.35	53.99	62.14	60.36	62.14	65.71	72.70	69.01	96.88	97.18	100.00	89.08	86.62	80.63	79.58	78.52	78.52	79.58	82.04	84.09	83.45	81.34	80.99	81.39	81.34	82.04	82.04	82.04	81.39	82.39	81.69	82.04
Aedes aegypti	53.26	58.33	57.61	62.14	60.50	62.14	65.71	70.57	70.53	90.23	89.44	89.08	100.00	94.04	79.93	78.87	77.46	78.17	78.87	81.34	84.09	82.04	80.28	80.28	80.29	80.99	80.63	80.63	80.63	79.93	81.34	80.99	81.34
Chironomus kiiensis	51.45	56.16	55.80	61.07	58.72	60.36	64.64	70.92	69.12	87.50	86.97	86.62	94.04	100.00	77.82	76.76	75.70	76.41	77.46	80.28	82.20	80.28	78.52	78.52	78.47	79.23	78.87	78.87	78.87	78.10	78.87	78.87	79.23
Dermatophagoides farinae	55.80	58.33	58.33	65.00	61.79	63.21	66.07	63.82	74.65	79.30	80.63	80.63	79.93	77.82	100.00	98.24	95.77	95.77	96.48	83.45	83.33	83.45	80.63	80.99	81.39	80.99	81.69	81.69	81.69	81.39	81.69	81.69	82.04
Dermatophagoides pteronyssinus	55.07	57.61	57.61	63.57	60.36	61.79	64.64	64.89	73.24	78.52	79.58	79.58	78.87	76.76	98.24	100.00	96.13	94.37	94.72	82.04	81.82	82.04	79.23	79.58	80.29	79.58	80.28	80.28	80.28	80.29	80.28	80.28	80.63
Lepidoglyphus destructor	56.52	57.61	57.97	63.57	60.71	62.14	64.29	63.83	73.59	76.95	78.52	78.52	77.46	75.70	95.77	96.13	100.00	96.13	95.77	80.99	79.92	80.28	78.87	79.58	80.29	79.58	80.28	80.28	80.28	80.29	79.93	79.93	80.63
Blomia tropicalis	55.80	58.33	58.70	64.64	61.79	63.21	65.36	63.83	74.30	76.95	78.52	78.52	78.17	76.41	95.77	94.37	96.13	100.00	96.48	80.99	80.30	80.63	78.87	79.58	79.93	79.58	80.28	80.28	80.28	79.93	79.93	79.93	80.28
Chortoglyphus arcuatus	55.80	57.97	58.33	64.64	62.14	63.57	66.43	64.89	74.65	78.12	79.58	79.58	78.87	77.46	96.48	94.72	95.77	96.48	100.00	82.04	81.06	81.34	79.93	80.63	81.02	80.63	81.34	81.34	81.34	81.02	80.99	80.99	81.69
Homarus americanus	54.71	58.33	57.61	63.93	61.43	62.86	68.21	69.86	74.65	81.25	83.10	82.04	81.34	80.28	83.45	82.04	80.99	80.99	82.04	100.00	96.59	97.18	92.96	93.31	93.80	93.31	93.31	93.31	93.31	93.07	93.31	93.31	93.31
Charybdis feriatus	56.64	60.55	59.77	61.92	59.62	61.54	68.85	69.96	74.24	82.42	84.09	84.09	84.09	82.20	83.33	81.82	79.92	80.30	81.06	96.59	100.00	99.62	90.53	91.29	91.34	91.67	91.67	91.67	91.67	91.34	91.29	91.29	91.29
Scylla paramamosain	54.71	58.70	57.97	63.57	61.07	62.86	68.21	70.57	75.00	82.81	83.10	83.45	82.04	80.28	83.45	82.04	80.28	80.63	81.34	97.18	99.62	100.00	91.55	91.90	92.34	91.90	92.61	92.61	92.61	92.34	92.25	92.25	92.25
Melicertus latisulcatus	54.35	58.70	57.61	64.64	62.14	63.93	68.21	69.86	71.83	80.47	82.04	81.34	80.28	78.52	80.63	79.23	78.87	78.87	79.93	92.96	90.53	91.55	100.00	94.01	95.26	94.72	95.07	95.07	95.07	94.89	94.72	94.72	95.42
Procambarus clarkii	54.35	57.97	57.61	63.93	61.07	62.50	66.79	69.15	72.18	80.08	82.04	80.99	80.28	78.52	80.99	79.58	79.58	79.58	80.63	93.31	91.29	91.90	94.01	100.00	97.81	97.18	96.83	96.83	96.83	96.72	95.42	96.13	94.72
Panulirus stimpsoni	53.01	56.77	56.39	63.33	61.11	62.59	67.41	71.32	71.90	80.49	82.12	81.39	80.29	78.47	81.39	80.29	80.29	79.93	81.02	93.80	91.34	92.34	95.26	97.81	100.00	98.54	98.18	98.18	98.18	98.18	96.72	97.45	95.99
Portunus pelagicus	53.62	57.61	57.25	63.57	60.71	62.14	67.86	69.15	72.18	81.25	82.04	81.34	80.99	79.23	80.99	79.58	79.58	79.58	80.63	93.31	91.67	91.90	94.72	97.18	98.54	100.00	97.54	97.54	97.54	97.45	96.13	96.83	95.42
Litopenaeus vannamei	53.62	57.97	57.61	63.93	61.07	62.86	68.21	69.86	72.89	81.25	82.39	82.04	80.63	78.87	81.69	80.28	80.28	80.28	81.34	93.31	91.67	92.61	95.07	96.83	98.18	97.54	100.00	100.00	100.00	99.64	97.54	98.24	96.83
Penaeus aztecus	53.62	57.97	57.61	63.93	61.07	62.86	68.21	69.86	72.89	81.25	82.39	82.04	80.63	78.87	81.69	80.28	80.28	80.28	81.34	93.31	91.67	92.61	95.07	96.83	98.18	97.54	100.00	100.00	100.00	99.64	97.54	98.24	96.83
Penaeus monodon	53.62	57.97	57.61	63.93	61.07	62.86	68.21	69.86	72.89	81.25	82.39	82.04	80.63	78.87	81.69	80.28	80.28	80.28	81.34	93.31	91.67	92.61	95.07	96.83	98.18	97.54	100.00	100.00	100.00	99.64	97.54	98.24	96.83
Metapenaeus ensis	53.63	56.77	56.39	62.96	60.37	62.22	67.41	71.69	71.90	80.49	81.75	81.39	79.93	78.10	81.39	80.29	80.29	79.93	81.02	93.07	91.34	92.34	94.89	96.72	98.18	97.45	99.64	99.64	99.64	100.00	97.45	98.18	96.72
Crangon crangon	54.35	58.70	57.61	63.93	61.79	63.21	68.57	69.86	73.24	81.64	82.75	82.39	81.34	78.87	81.69	80.28	79.93	79.93	80.99	93.31	91.29	92.25	94.72	95.42	96.72	96.13	97.54	97.54	97.54	97.45	100.00	98.94	98.59
Macrobrachium rosenbergii	54.35	58.70	57.61	64.29	61.43	62.86	68.57	69.86	73.24	80.86	82.04	81.69	80.99	78.87	81.69	80.28	79.93	79.93	80.99	93.31	91.29	92.25	94.72	96.13	97.45	96.83	98.24	98.24	98.24	98.18	98.94	100.00	98.59
Pandalus borealis	55.43	59.42	58.33	64.29	61.79	63.21	68.93	69.50	73.24	81.25	82.39	82.04	81.34	79.23	82.04	80.63	80.63	80.28	81.69	93.31	91.29	92.25	95.42	94.72	95.99	95.42	96.83	96.83	96.83	96.72	98.59	98.59	100.00

Supplementary Figure 1. Percent identity matrix comparing tropomyosin allergens listed in the official Allergen Nomenclature Database (www.allergen.org) with tropomyosin from bed bugs.

Supplementary Table 1. Bed bug tropomyosin data

Sample	Sample type	ng tropomyosin/sample	Sample	Sample type	ng tropomyosin/sample	Sample	Sample type	ng tropomyosin/sample
F1	Feces	0.00	H101	Aged, 1 month	348.60	HC1	Cimexa killed	56.42
F3	Feces	0.00	H102	Aged, 1 month	461.70	HC2	Cimexa killed	94.07
F4	Feces	0.00	H103	Aged, 1 month	174.36	HC3	Cimexa killed	40.68
F5	Feces	0.00	H104	Aged, 1 month	372.20	HC4	Cimexa killed	65.58
F7	Feces	0.00	H105	Aged, 1 month	245.30	HC5	Cimexa killed	42.34
F10	Feces	0.00	H106	Aged, 1 month	287.35	HC6	Cimexa killed	73.37
WI1	Whole body, intact	0.00	H107	Aged, 1 month	674.00	HH1	Heat killed	294.80
WI2	Whole body, intact	0.00	H108	Aged, 1 month	407.40	HH2	Heat killed	329.05
WI3	Whole body, intact	0.00	H109	Aged, 1 month	675.87	HH3	Heat killed	391.95
WI4	Whole body, intact	0.00	H110	Aged, 1 month	649.80	HH4	Heat killed	318.15
WI5	Whole body, intact	0.00	H301	Aged, 3 months	468.30	HH5	Heat killed	308.10
WI6	Whole body, intact	0.00	H302	Aged, 3 months	189.69	HH6	Heat killed	333.80
WI7	Whole body, intact	0.00	H303	Aged, 3 months	261.70			
WI8	Whole body, intact	0.00	H304	Aged, 3 months	308.30			
WI9	Whole body, intact	0.00	H305	Aged, 3 months	438.55			
WI10	Whole body, intact	0.00	H306	Aged, 3 months	330.40			
E1	Exuviae	0.02	H307	Aged, 3 months	681.70			
E2	Exuviae	0.01	H308	Aged, 3 months	270.40			
E3	Exuviae	0.00	H309	Aged, 3 months	193.31			
E4	Exuviae	0.01	H310	Aged, 3 months	522.60			
E5	Exuviae	0.01	H601	Aged, 6 months	954.80			
E6	Exuviae	0.00	H604	Aged, 6 months	623.80			
E7	Exuviae	0.00	H605	Aged, 6 months	397.65			
E8	Exuviae	0.00	H606	Aged, 6 months	627.60			
E9	Exuviae	0.00	H607	Aged, 6 months	449.70			
WF1	Whole body, fragmented	221.02	H608	Aged, 6 months	396.10			
WF2	Whole body, fragmented	246.40	H609	Aged, 6 months	329.30			
WF3	Whole body, fragmented	175.46	H610	Aged, 6 months	446.45			
WF4	Whole body, fragmented	176.74	H611	Aged, 6 months	750.95			
WF5	Whole body, fragmented	118.58	H612	Aged, 6 months	542.65			
WF6	Whole body, fragmented	213.21	H1801	Aged, 18 months	393.60			
WF7	Whole body, fragmented	131.10	H1802	Aged, 18 months	96.29			
WF8	Whole body, fragmented	240.73	H1803	Aged, 18 months	132.08			
WF9	Whole body, fragmented	487.75	H1804	Aged, 18 months	218.45			
			H1805	Aged, 18 months	78.94			
			H1807	Aged, 18 months	242.00			
